# Associations between plasma metabolism-associated proteins and future development of giant cell arteritis: results from a prospective study

**DOI:** 10.1093/rheumatology/keae073

**Published:** 2024-02-03

**Authors:** Karin Wadström, Lennart T H Jacobsson, Aladdin J Mohammad, Kenneth J Warrington, Eric L Matteson, Magnus E Jakobsson, Carl Turesson

**Affiliations:** Rheumatology, Department of Clinical Sciences, Lund University, Malmö, Sweden; Center for Rheumatology, Academic Specialist Center, Region Stockholm, Stockholm, Sweden; Rheumatology, Department of Clinical Sciences, Lund University, Malmö, Sweden; Department of Rheumatology & Inflammation Research, Institute of Medicine, The Sahlgrenska Academy, University of Gotherburg, Gothenburg, Sweden; Department of Rheumatology, Skåne University Hospital, Malmö, Sweden; Department of Medicine, University of Cambridge, Cambridge, UK; Division of Rheumatology, Mayo Clinic College of Medicine and Science, Rochester, MN, USA; Division of Rheumatology, Mayo Clinic College of Medicine and Science, Rochester, MN, USA; Department of Biomedical Science, Faculty of Health and Society, Malmö University, Malmö, Sweden; Rheumatology, Department of Clinical Sciences, Lund University, Malmö, Sweden; Department of Rheumatology, Skåne University Hospital, Malmö, Sweden

**Keywords:** giant cell arteritis, biomarkers, metabolism, macrophage activation, pathogenesis

## Abstract

**Objective:**

The aim of this study was to investigate the relationship between biomarkers associated with metabolism and subsequent development of GCA.

**Method:**

Participants in the population-based Malmö Diet Cancer Study (MDCS; *N* = 30 447) who were subsequently diagnosed with GCA were identified in a structured process. Matched GCA-free controls were selected from the study cohort. Baseline plasma samples were analysed using the antibody-based OLINK proteomics metabolism panel (92 metabolic proteins). Analyses were pre-designated as hypothesis-driven or hypothesis-generating. In the latter, principal component analysis was used to identify groups of proteins that explained the variance in the proteome.

**Results:**

There were 95 cases with a confirmed incident diagnosis of GCA (median 12.0 years after inclusion). Among biomarkers with a priori hypotheses, adhesion G protein-coupled receptor E2 (ADGRE2) was positively associated [odds ratio (OR) per S.D. 1.67; 95% CI 1.08–2.57], and fructose-1,6-bisphosphatase 1 (FBP1) was negatively associated (OR per S.D. 0.59; 95% CI 0.35–0.99) with GCA. In particular, ADGRE2 levels were associated with subsequent GCA in the subset sampled <8.5 years before diagnosis. For meteorin-like protein (Metrnl), the highest impact on the risk of GCA was observed in those patients sampled closest to diagnosis, with a decreasing trend with longer time to GCA (*P* = 0.03). In the hypothesis-generating analyses, elevated levels of receptor tyrosine-like orphan receptor 1 (ROR1) were associated with subsequent GCA.

**Conclusion:**

Biomarkers identified years before clinical diagnosis indicated a protective role of gluconeogenesis (FBP1) and an association with macrophage activation (ADGRE2 and Metrnl) and proinflammatory signals (ROR1) for development of GCA.

Rheumatology key messages

- This study indicates activation of the innate immune system in the preclinical phase of GCA.
- Plasma metabolism-related protein patterns are compatible with early macrophage activation and polarization in GCA.
- Insights into early key mechanisms may be useful for developing new treatment strategies for GCA.

## Introduction

GCA is the most common large-vessel vasculitis in persons aged >50 years in the western world, affecting medium- to large-sized arteries, with a female predominance [1]. The highest incidences have been reported from Scandinavian countries and Minnesota, USA, which have rates of ∼20–30/100 000 among persons aged over 50 years [2, 3].

Several factors that may predict GCA years before clinical onset have been described. In a nested case–control study from our group, individuals who subsequently developed GCA had lower blood glucose, cholesterol and triglycerides at baseline, a median of 21 years before disease onset, compared with controls [4]. Similar results have been reported in a retrospective survey of another population [5]. Several studies have found an association between low BMI prior to diagnosis and subsequent GCA [6–8]. In addition to a lower BMI, a retrospective case–control study from Gothenburg found that current smoking and multiple hormone-related factors were associated with increased risk of GCA [6].

Incident GCA cases have been identified as having a significantly lower prevalence of diabetes mellitus compared with controls at the time of diagnosis [9]. Taken together, subsequent GCA cases seem to have better metabolic control and a lower BMI, and to be less likely to suffer from diabetes prior to diagnosis.

Furthermore, T cell checkpoint dysregulation may play a part in the pathogenesis of GCA, as a higher expression of programmed cell death receptor-1 (PD-1) on T cells and lower expression of programmed death ligand-1 (PD-L1) on dendritic cells (DCs) have been reported in temporal artery biopsies (TABs) from GCA patients [10]. PD-1/PD-L1 interaction normally leads to inhibition of the T cell receptor activating cascade, resulting in an attenuated immune response [11]. A connection between the PD1/PD-L1 checkpoint and glucose metabolites has been proposed, as a study by Watanabe *et al.* showed a positive association between mitochondrial pyruvate and the expression of PD-L1 on macrophages [12]. This indicates that glucose levels might play a part in the pathogenesis of GCA.

In a previous study with a similar design, our group found that some biomarkers associated with inflammation were elevated in pre-GCA cases compared with controls [13]. In particular, elevated levels of IFN-γ and MCP3 were found years prior to diagnosis in individuals who subsequently developed GCA. Several other proteins known to be important for T cell function were also associated with GCA in these analyses, e.g. CXCL9, IL-2, CD40 and CCL25**.** These results suggest that activation of the adaptive immune system may precede the clinical onset [13].

In this study, we aimed to investigate metabolism-associated plasma proteins prior to onset of GCA. To our knowledge, this has not been done previously.

## Material and methods

### Source population and exposure information

The Malmö Diet Cancer Study (MDCS) is a community-based health survey performed in Malmö in 1991–1996. All women born 1923–50 and all men born 1923–45 who were residents of Malmö were invited to participate. Exclusion criteria were insufficient Swedish language skills and mental incapacity. The total source population was 74 138 persons, and the participation rate was 40.8%. A total of 30 447 participants (12 121 men and 18 326 women) were included. The mean age at screening was 58 years in women and 59 years in men. Using a self-administered questionnaire, information on lifestyle factors and current health status was collected from all participants. Non-fasting blood samples were obtained at the time of inclusion in the health survey in a standardized manner and stored at –80°C. Further details on the MDCS are described elsewhere [14].

### Cases and controls

All cases had participated in the MDCS prior to being diagnosed with GCA. Patients were identified based on ICD diagnosis codes indicating GCA in the local outpatient clinic administrative register for Malmö University Hospital or the National Patient Register through 31 December 2011. A structured review of medical records was performed, and cases were classified according to the 1990 ACR criteria for GCA [15]. Some cases with typical clinical features were included, based on expert opinion, even if they did not fulfil the classification criteria. In addition, data on visual manifestations, initial dose of glucocorticoids, large-vessel involvement, and other disease characteristics were collected.

One control for each validated case who was alive and free from GCA when the index person was diagnosed with GCA was randomly selected from the MDCS. The controls were matched for sex, year of birth and year of screening.

We identified 100 cases with corresponding controls. After excluding those who had no preserved plasma samples, or insufficient sample volume, data were available for 95 cases and 97 controls.

The regional research ethics committee for southern Sweden approved the study (registration number 308/2007). When included in the MDCS, all participants gave their written informed consent for future use of their collected information and samples for research purposes. No additional consent for participation in this study was obtained. Neither patients nor the public were involved in the study design, recruitment, or dissemination of results.

### Plasma proteomic biomarkers

A large panel with 92 metabolic proteins (Olink^®^ Metabolic panel [16]) was used to investigate potential biomarkers associated with metabolism prior to clinical disease onset in patients developing GCA. All biomarkers analysed are presented in Supplementary Table S1, available at *Rheumatology* online.

Plasma levels of proteins were analysed by the Proximity Extension Assay (PEA) technique, using a multiplex reagent kit (O-link Bioscience, Uppsala, Sweden), and the analysis has been described in detail elsewhere [17]. The results are presented with arbitrary units. As the platform provides relative protein quantification as log2 normalized protein expression (NPX), every unit increase corresponds to a doubling in the relative protein concentration.

### Statistics

The statistical analyses were specified in a study protocol written before obtaining the data. The analyses were separated into two categories, and analyses involving biomarkers with an a priori hypothesis formulated by the authors were handled separately from analyses involving all biomarkers. The latter were regarded as hypothesis-generating analyses.

Variables that were not normally distributed were log-transformed using the natural logarithm. Normality of distribution was assessed using visual inspection of histograms and the Shapiro-Wilk test. Eight biomarkers, all with Shapiro-Wilk statistics of <0.85, were log-transformed. To allow for logarithmic computation without censoring individuals with negative NPX values, the smallest possible constant was added to the arbitrary values.

### Biomarkers with a priori hypotheses

Six biomarkers were selected for evaluation of a priori hypotheses biomarkers (Supplementary Table S2, available at *Rheumatology* online). Five of them were assumed to be elevated: meteorin-like protein (Metrnl), fructose-1,6-bisphosphatase 1 (FBP1), galanin peptides (GAL), adhesion G protein-coupled receptor E2 (ADGRE2), nectin-2, and one was assumed to be reduced: appetite-regulating hormone ghrelin (GHRL). These proteins were selected based on previous knowledge of function and/or association with other inflammatory diseases [18–24].

To examine potential biomarker predictors, we used conditional logistic regression, with case status as the outcome. A group number connecting each case and its corresponding control was entered in the logistic regression models as a categorical variable. Odds ratios (ORs) were calculated per S.D. to enable comparisons of effect sizes.

Further, the analyses were stratified by time from screening to GCA diagnosis (by quartiles) in years. Associations across quartiles (*P* for trend) were assessed by examining the interactions between quartile of time to diagnosis and biomarker levels in separate logistic regression models. Multiple hypothesis testing was handled using the Holm correction approach [25], and both corrected and original *P*-values are presented.

### Hypothesis-generating analyses

To identify groups of protein that explained variance in the proteome, we used principal component analysis (PCA). Before inclusion in the PCA, *z*-scores were computed for all biomarkers to enable comparability. Assumptions were fulfilled for the Kaiser–Meyer–Olkin (KMO) test for sampling adequacy and Bartlett’s test for sphericity, respectively. We set the preliminary cut-off for the Eigenvalue to >2.0 to reduce the number of components, resulting in seven components. Of these, components 5–7 were excluded from the analyses of associations between individual contributing proteins and GCA, based on limited numbers of variables with high factor loading (Supplementary Table S3, available at *Rheumatology* online) and because they were considered to add minor contributions based on the scree plot (Supplementary Fig. S1, available at *Rheumatology* online).

The seven components were included as independent variables in logistic regression models evaluating their relation to the risk of subsequent GCA.

In the next step, the biomarkers with a loading of >0.7 within the selected components [1–4] were investigated using the same statistical protocol as for the a priori analyses, except that correction for multiple testing was not applied to the hypothesis-generating approach. All statistical analyses were performed using the Statistical Package for Social Sciences (version 24.0; IBM, Armonk, NY, USA).

## Results

### Incident cases

A total of 95 cases were identified with a confirmed incident diagnosis of GCA and available results from the plasma proteome analyses. Of the total cohort, 78 (82%) of the cases were female, 64% had a positive temporal artery biopsy (TAB) and 90.5% fulfilled the ACR 1990 classification criteria for GCA [15]. At the time of diagnosis, the median age was 73.5 years (range 56.1–85.8) and the median time from screening to diagnosis was 12.0 years (range 0.3–19.1) (Table 1). Only one case was screened <1 year before diagnosis.

**Table 1.** Characteristics of patients with GCA at diagnosis

	Cases	Controls
***N***	95	97
**Female sex**	78 (82%)	78 (80%)
**Age at screening**	62.1 (S.D. 6.7; range 47.0–73.2)	61.2 (S.D. 6.7; range 47.0–72.5)
**Time from screening to index date** ^a^ **, years, median**	12.0 (range 0.3–19.1)	11.5 (range 0.5–18.2)
**Age at GCA diagnosis, mean, years**	73.5 (S.D. 6.1; range 56.9–85.8)	N/A
**Biopsy positive**	61 (64.2%)	N/A
**Fulfilled ACR classification criteria for GCA**	86 (90.5%)	N/A
**Visual impairment at diagnosis**	40 (42.1%)	N/A
**Large-vessel involvement**	13 (14%)	N/A
**ESR at diagnosis (mm/h) (mean)** ^b^	80 (S.D. 31.4)	N/A
**CRP at diagnosis (median)** ^b^	86 (IQR 49–141)	N/A
**Initial glucocorticoid dose (mg prednisolone) (median)** ^c^	40 (IQR 40–60)	N/A

a Date of GCA diagnosis and corresponding date in the matched control.

b Missing data: ESR *n* = 9, CRP *n* = 25.

c Eight patients initially treated with i.v. glucocorticoids. IQR: interquartile range.

Proportions with a self-reported history of comorbidities or anti-hypertensive treatment at the time of screening were similar in cases and controls. No cases or controls were treated with glucocorticoids, and <10% were on anti-diabetic or lipid-lowering drugs. (Supplementary Tables S4 and S5, available at *Rheumatology* online).

### Testing of a priori hypotheses

ADGRE2 was higher in cases compared with controls [(mean (NPX) 4.88 *vs* 4.79] (Table 2), [OR (per S.D.) 1.67; 95% CI 1.08–2.57, *P* = 0.022)]. In analysis stratified by time from screening to diagnosis (quartiles), the highest OR was found in the subset sampled closer to GCA diagnosis (0.32–8.49 years) (OR 3.91; 95% CI 1.45–10.60). Although the wide CI indicates uncertainty, the association in this subset was significant even after correction for multiple testing (*P* = 0.042). There was a decreasing trend by quartile 3 (Table 3), but the trend over all four quartiles did not reach significance (*P* = 0.257).

**Table 2.** Baseline values for biomarkers with a priori hypotheses, in subsequent GCA cases compared with controls

	All		Women		Men	
	Cases (95)	Controls (97)	**Cases (**78**)**	**Controls (**78**)**	**Cases (**17**)**	**Controls (**19**)**
**Metrnl**	3.44 (0.26)	3.41 (0.27)	3.45 (0.25)	3.41 (0.26)	3.40 (0.30)	3.40 (0.30)
**FBP1**	0.95 (0.79)	1.10 (0.80)	0.96 (0.80)	1.09 (0.80)	0.88 (0.75)	1.14 (0.83)
**GAL**	5.04 (0.61)	5.06 (0.89)	5.00 (0.60)	4.99 (0.90)	5.25 (0.64)	5.37 (0.77)
**GHRL**	2.33 (0.87)	2.39 (0.88)	2.45 (0.84)	2.47 (0.84)	1.77 (0.78)	2.06 (0.94)
**ADGRE2**	4.88 (0.37)	4.79 (0.38)	4.87 (0.36)	4.77 (0.32)	4.91 (0.43)	4.89 (0.57)
**NECTIN2**	5.72 (0.35)	5.67 (0.37)	5.70 (0.34)	5.68 (0.37)	5.81 (0.38)	5.65 (0.36)

All values are in arbitrary units, NPX. Means (S.D.). NPX: log2 normalized protein expression; ADGRE2: adhesion G protein-coupled receptor E2; FBP1: fructose-1,6-bisphosphatase 1; GAL: galanin peptides; GHRL: ghrelin; Metrnl: meteorin-like protein.

**Table 3.** Potential biomarkers of subsequent GCA, overall and stratified for time from screening to diagnosis

	All			Quartile 1 (0.32–8.49) (*N*^a^ = 45)			Quartile 2 (8.50–11.96) (*N*^a^ = 49)			Quartile 3 (11.97–15.47) (*N*^a^ = 46)			Quartile 4 (15.48–19.11) (*N*^a^ = 48)			
	OR (CI)	*P*	*P* (corr)	OR (CI)	*P*	*P* (corr)	OR (CI)	*P*	*P* (corr)	OR (CI)	*P*	*P* (corr)	OR (CI)	*P*	*P* (corr)	*P* for trend*
Metrnl	1.42 (0.90–2.23)	0.14	0.54	2.40 (0.98–5.85)*	0.05*	0.28	3.13 (0.92–10.6)*	0.07*	0.40	0.90 (0.38–2.15)	0.82	1.00	0.74 (0.29–1.89)	0.53	1.00	0.03*
FBP1	0.59 (0.35–0.99)*	0.04*	0.22	0.29 (0.06–1.39)	0.12	0.36	0.84 (0.43–1.65)	0.61	1.00	1.13 (0.38–3.30)	0.83	1.00	0.09 (0.02–0.48)*	0.005*	0.03	0.24
GAL	0.98 (0.66–1.45)	0.92	0.92	2.32 (0.83–6.51)	0.11	0.44	0.65 (0.29–1.49)	0.31	1.00	0.38 (0.15–0.97)	0.04	0.22	2.17 (0.92–5.13)*	0.08*	0.38	0.98
GHRL	0.83 (0.55–1.25)	0.38	0.75	1.20 (0.52–2.78)	0.68	0.68	1.38 (0.66–2.88)	0.39	1.00	0.39 (0.16–0.96)	0.04	0.25	0.55 (0.20–1.48)	0.23	0.70	0.06*
ADGRE2	1.67 (1.08–2.57)*	0.02*	0.13	3.91 (1.45–10.6)*	0.007*	0.04*	1.17 (0.52–2.64)	0.71	0.71	0.94 (0.44–2.00)	0.86	0.86	2.90 (0.83–10.2)	0.10	0.38	0.26
NECTIN2	1.33 (0.88–2.01)	0.18	0.53	1.47 (0.66–3.32)	0.35	0.70	1.64 (0.71–3.82)	0.25	0.24	1.40 (0.63–3.11)	0.41	1.00	0.84 (0.34–2.09)	0.71	0.71	0.38

a Total of cases and controls. Conditional logistic regression analysis of biomarkers with a priori hypotheses. Odds ratios (ORs) per S.D. with 95% CIs. *P*-values with and without Holm correction for multiple testing. *P < 0.10. ADGRE2: adhesion G protein-coupled receptor E2; FBP1: fructose-1,6-bisphosphatase 1; GAL: galanin peptides; GHRL: ghrelin; Metrnl: meteorin-like protein.

FBP1 levels were lower in cases compared with controls [(mean (NPX) 0.95 *vs* 1.10] (Table 2), [OR (per S.D.) 0.59; 95% CI 0.35–0.99, *P* = 0.044)). In the analysis stratified by time from screening to diagnosis, the lowest OR was found in the quartile sampled closer to GCA diagnosis (OR 0.29; 95% CI 0.06–1.39), with an increasing trend by quartile three.

Overall, there were no significant difference between levels of Metrnl in cases compared with controls [OR (per S.D.) 1.42; 95% CI 0.90–2.23, *P* = 0.135]. However, in the stratified analysis there was a significant trend (*P* = 0.030), with higher ORs in those sampled closer to GCA diagnosis, i.e. quartile 1 [OR (per S.D.) 2.40; 95% CI 0.98–5.85, *P* = 0.055] and quartile 2 [8.50–11.96 years before diagnosis; OR (per S.D.) 3.13; 95% CI 0.92–10.63, *P* = 0.067], respectively, compared with those sampled with a longer duration to diagnosis. For GAL, GHRL and nectin-2, there were no significant associations with subsequent development of GCA.

### Hypothesis-generating results

For the seven components with Eigenvalues above 2.0 in the PCA, factor loadings for every protein are shown in Supplementary Table S3, available at *Rheumatology* online. Descriptive data for these components are shown in Table 4. They were further investigated as potential predictors of GCA, using each component as an individual variable in logistic regression models. Component number 4 significantly predicted subsequent GCA (OR 2.06; 95% CI 1.21–3.49, *P* = 0.008) (Table 4). With an Eigenvalue of 3.88, component number 4 explained 4.21% of the variance in the proteome, which was mainly driven by FBP1 which was the only protein with a factor loading of >0.7 within this component (Supplementary Table S3, available at *Rheumatology* online). Components 1–4 included 26 variables that met the factor loading cut-off and were therefore further investigated (Table 5).

**Table 4.** Principal component analysis component characteristics, and relation to risk of GCA (conditional logistic regression)

PCA			Logistic regression	
	Eigenvalue	% of variance	OR (95% CI)	*P* value
Component 1	26.28	28.56	1.04 (0.64–1.68)	0.87
Component 2	9.53	10.35	1.25 (0.78–2.02)	0.36
Component 3	3.90	4.24	0.96 (0.63–1.48)	0.86
Component 4	3.88	4.21	2.06 (1.21–3.49)*	0.008*
Component 5	2.56	2.78	1.00 (0.66–1.52)	0.98
Component 6	2.29	2.49	1.03 (0.69–1.54)	0.89
Component 7	2.03	2.21	1.15 (0.73–1.82)	0.56

* *P* < 0.10. PCA: principal component analysis.

**Table 5.** Biomarkers with a loading of >0.7 in PCA, baseline values, and relation to GCA risk

	Baseline values (NPX)		Conditional logistic regression	
	Cases (*n* = 95)	Controls (*n* = 97)	OR (95% CI)	*P* value
**PCA 1**				
ENO2	3.01 (0.54)	3.01 (0.71)	1.42 (0.83–2.43)	0.20
SERPINB6	2.67 (0.42)	2.60 (0.61)	1.36 (0.84–2.20)	0.21
DIABLO	2.10 (0.74)	2.08 (0.91)	0.95 (0.62–1.46)	0.82
CA13	3.45 (1.00)	3.27 (1.17)	1.65 (0.96–2.82)	0.07
GRAP2	1.28 (0.44)	1.30 (0.58)	0.85 (0.54–1.33)	0.47
ANXA11	–0.08 (0.74)	–0.10 (0.87)	0.99 (0.65–1.49)	0.95
QDPR	3.91 (0.46)	3.90 (0.56)	0.97 (0.61–1.52)	0.88
SNAP23	1.90 (0.70)	1.87 (0.84)	1.06 (0.66–1.69)	0.82
COMT	2.47 (0.76)	2.46 (0.88)	0.98 (0.63–1.51)	0.92
CRKL	6.68 (0.97)	6.60 (1.20)	1.12 (0.69–1.81)	0.65
DAB2	0.89 (0.33)	0.90 (0.43)	0.99 (0.63–1.54)	0.95
ANXA4	0.48 (0.31)	0.49 (0.39)	1.03 (0.67–1.58)	0.89
**PCA 2**				
Metrnl	3.44 (0.26)	3.41 (0.27)	1.42 (0.90–2.23)	0.14
NECTIN2	5.72 (0.35)	5.67 (0.37)	1.33 (0.88–2.01)	0.18
CLIMP	2.71 (0.44)	2.76 (0.51)	0.74 (0.48–1.15)	0.18
SEMA3F	2.38 (0.28)	2.36 (0.29)	1.12 (0.74–1.70)	0.59
CCDC80	5.83 (0.52)	5.76 (0.50)	1.43 (0.89–2.29)	0.14
FAM3C	6.29 (0.47)	6.25 (0.45)	1.15 (0.74–1.79)	0.97
LRP11	4.06 (0.40)	4.01 (0.38)	1.33 (0.87–2.04)	0.19
CANT1	4.64 (0.28)	4.60 (0.26)	1.36 (0.89–2.07)	0.16
IGFBPL1	3.12 (0.35)	3.15 (0.41)	0.78 (0.48–1.27)	0.78
NPDC1	5.39 (0.38)	5.34 (0.35)	1.48 (0.93–2.37)	0.10
ROR1	2.53 (0.39)	2.42 (0.42)	1.61 (1.05–2.46)*	0.03*
**PCA 3**				
CD2AP	4.10 (0.58)	4.10 (0.71)	0.93 (0.62–1.40)	0.74
HDGF	0.17 (0.57)	0.14 (0.54)	1.02 (0.69–1.50)	0.94
**PCA 4**				
FBP1	0.95 (0.79)	1.10 (0.82)	0.59 (0.35–0.99)*	0.04*

Baseline values are in arbitrary units, NPX. Mean (S.D.).

* *P* < 0.10. PCA: principal component analysis; FBP1: fructose-1,6-bisphosphatase 1; Metrnl: meteorin-like protein; ROR1: receptor tyrosine-like orphan receptor 1.

Of these 26 variables, 2 revealed significant association per S.D. with subsequent GCA (Table 4). FBP1, found to be significant in the a priori hypothesis testing described above, was also identified as a potential predictor of GCA through the hypothesis-generating analysis. In addition, higher ROR1 concentrations were associated with increased risk of GCA (OR per S.D. 1.61; 95% CI 1.05–2.46). Stratification by time from screening to diagnosis for FBP1 is described above, and for ROR1 no significant trend depending on sampling time was observed (Table 6).

**Table 6.** Conditional logistic regression for hypothesis generating biomarkers, stratified for time from screening to diagnosis (years)

	Quartile 1 (0.3–8.5) (*N*^a^ = 45)		Quartile 2 (8.5–11.9) (*N*^a^ = 49)		Quartile 3 (11.9–15.5) (*N*^a^ = 46)		Quartile 4 (15.5–19.1) (*N*^a^ = 48)		
	OR (CI)	*P* value	OR (CI)	*P* value	OR (CI)	*P* value	OR (CI)	*P* value	*P* for trend^b^
ROR1	1.39 (0.58–3.32)	0.46	1.95 (0.95–4.03)*	0.07*	1.08 (0.44–2.67)	0.87	2.08 (0.76–5.67)	0.15	0.85
FBP1	0.29 (0.06–1.39)	0.12	0.84 (0.43–1.65)	0.61	1.13 (0.38–3.30)	0.83	0.09 (0.02–0.48)*	0.005*	0.24

a Total of cases and controls.

b Based on interaction between quartile and levels of biomarkers, using logistic regression models. Odds ratios (ORs) per S.D. with 95% CIs.

* *P* < 0.10. ROR1: receptor tyrosine-like orphan receptor 1; FBP1: fructose-1,6-bisphosphatase 1.

## Discussion

In this study, we investigated proteins selected on the basis of known association with metabolism. Our results indicated that a subset of these biomarkers was elevated or decreased in subsequent GCA cases compared with controls, indicating that these proteins might play a part in GCA pathogenesis and should be further investigated. We found that levels of ADGRE2 were significantly higher in cases compared with controls, overall, and in particular in those sampled close to diagnosis, with a decreasing trend for increased time to diagnosis. Metrnl also had higher ORs in those sampled closer to diagnosis, with a significantly decreasing trend in those sampled with a longer time duration to diagnosis.

Metrnl was initially identified as a protein with neurotrophic functions [26]. Thereafter it was described as an adipokine produced by white adipose tissue, with a potential role in insulin sensitization [26]. However, in clinical studies on the relationship between circulating Metrnl levels and type 2 diabetes mellitus, there have been conflicting results [26, 27].

Recent data suggest a role for Metrnl in inflammation [27]. For example, associations between Metrnl and inflammatory diseases such as psoriasis, atopic dermatitis and RA have been described. A study comparing biopsies and samples from patients with RA, PsA and OA found higher levels of Metrnl in both the synovium and SF in RA and PsA [28].

More recently, further knowledge on Metrnl’s role in inflammation has come to light. It is expressed on activated macrophages, and *in vivo* Metrnl levels increase with inflammatory response [27, 29]. Knockout mice who do not express Metrnl exhibit multiple immune system abnormalities, for example lower levels of IgG in plasma, and downregulation of chemokine production [29].

ADGR2, also known as CD312 and EMR2, is a receptor initially identified as a myeloid-restricted transcript expressed in monocytes, macrophages, myeloid dendritic cells, and granulocytes [30]. Distinct myeloid populations have differential expression patterns of EMR2, suggesting a regulatory role in neutrophil function [18, 31]. Kuan-Yu *et al.* showed that the EMR2 receptor is a surface marker for macrophage differentiation, and that EMR2 activation eventually leads to MAPK and NF-kB signalling. Through this pathway, it induces expression of pro-inflammatory mediators, including IL-8, TNF-α and MMP9 [18]. Kop *et al.* found that EMR2 expression in the synovial sublining was significantly higher in RA patients compared with OA patients and ReA control patients. Most EMR2-positive cells were macrophages and dendritic cells [32].

ADGRE2/EMR2 signalling has also been implicated in ANCA-associated vasculitis (AAV). Irmscher *et al.* demonstrated that human serum factor H-related protein (FHR1) induces inflammasome NLRP3 *in vitro* through EMR2, independent of complement. This induction results in the secretion of pro-inflammatory cytokines such as IL-1β, TNF-α, IL-18 and IL-6. Furthermore, *in vitro* FHR1 selectively binds to necrotic cells in necrotic glomerulae of AAV patients, and in atherosclerotic plaques. Circulating FHR1 concentrations of AAV patients was correlated with levels of inflammation and progressive disease [33].

Taken together, both Metrnl and ADGRE2 are associated with macrophage activation, and expression and circulating levels seem to vary depending on degree of inflammation. They have independently been associated with other inflammatory diseases, such as RA. Macrophage polarization is an important feature in GCA pathogenesis [34], in which a distinct subset of CD206+ cells may stimulate tissue destruction and remodelling, through the production of MMP9 [35]; further studies of possible effects of these markers on macrophage activation and polarization in a GCA context would be of interest.

Levels of FBP1 were found to be significantly lower in cases compared with controls, overall, and with the lowest ORs in those sampled closest to diagnosis. FBP1 is a key enzyme in the glycolytic pathway [36]. It directly catalyses hydrolysis of fructose-1,6-bisphosphate to fructose-6-phosphate in gluconeogenesis, i.e. reversing the PFK1-catalysed phosphorylation of fructose-6-phosphate to fructose-1,6-bisphosphate in glycolysis.

Our results suggest that FBP1 may have some protective effect against GCA. FBP1 is mainly expressed in the kidneys and the liver and plays a critical role in maintaining blood glucose levels. In animal models, inhibition of FBP1 leads to inhibited gluconeogenesis and increased glucose sensitivity. An upregulation of FBPs (mainly FBP1) occurs in diabetes-susceptible obese mice [37], suggesting that it is important in type II diabetes mellitus (T2DM). Moreover, transgenic mice overexpressing human FBP1 specifically in pancreatic islet β-cells show reduced insulin secretion [38]. FBP1 inhibition has been investigated as a treatment for T2DM, but no such drug has been licensed to date [39]. Although animal model studies indicate that FBP provides negative feedback that limits weight gain [24], which, given the observed negative association between BMI and GCA [6–8], would argue for it as a biomarker of increased GCA risk, it may also be that FBP1 activity, leading to increased availability of pyruvate, downregulates PD-L1 in macrophages, a pathway that would be protective against GCA [10, 12]. As this pattern is against our original hypothesis, it should be interpreted with caution, and this new concept should be supported by additional studies and other experimental data.

In our hypothesis-generating analysis, the tyrosine kinase enzyme ROR1 was identified through PCA analysis and further found to be significantly higher in cases compared with controls. ROR1 is a receptor shown to be significant in embryonic development and cancer. In chronic lymphocytic leukaemia (CLL), a distinct expression of ROR1 on tumour cells has been identified, and ROR1 is currently one out of six markers recommended internationally for refining the diagnosis of CLL [40]. Surface expression of ROR1 has also been associated with other haematologic malignancies. When activated, ROR1 signals via transcription factors, including NF-κB [41]. The role of this pathway in the preclinical phase of GCA should be further explored.

The present findings, together with the previously reported upregulation of T cell–related proteins [13], suggest activation of a broad range of pro-inflammatory mechanisms in the preclinical phase of GCA. The potential roles of macrophage polarization and activation, potentially regulated by glycolysis, and NF-κB signalling, should be taken into account when developing treatment strategies aiming at early and sustained remission in patients with GCA.

Limitations of our study include the relatively small sample size, which affected the precision of the estimates, as shown by the CIs for the stratified analyses. Changes of biomarkers over time could not be assessed, as the samples were obtained at a single time point. It would be of interest to examine these markers over time, and to investigate how they relate to active vasculitis at the time of diagnosis.

The reported NPX values are reported in arbitrary units and are therefore not convertible to absolute concentrations. They can only be compared with the relative abundance of each biomarker across the sample cohort and should not be used for ranking the relative abundance of biomarkers within samples. This makes it difficult to compare the findings with those of previous studies on biomarkers in active disease.

Moreover, plasma levels of biomarkers do not necessarily reflect levels in relevant tissues, i.e. lymphoid organs or arteries [42]. As there are no previous studies on pre-diagnostic samples, except for the one from our group [13], the candidate biomarkers were selected, based on knowledge about established GCA, from within the group of biomarkers included in the OLINK panel.

Aspects of pre-analytic handling and analytic procedures can always be a source of bias.

Pre-analytical procedures for blood sample collection and storage, and storage time in the freezer may all be sources of bias. The risk of such bias was minimized by matching cases and controls at the time point for inclusion.

Our results can only be generalized to populations of mainly Scandinavian ethnicity in the investigated age groups. As no validation cohort was accessible in this study set-up, further investigations on this matter in other cohorts are needed to confirm generalizability.

Strengths of our study include the well-defined cohort, in which cases were validated through a structured review process, and the matching of controls on age and sex, which enabled comparisons of levels of biomarkers, as concentrations of various biomarkers may differ between the sexes and are known to change during the lifetime [43].

Another strength is the standardized manner in which the blood samples were obtained and stored, which was appropriate for later analysis.

Mass spectrometry (MS) would have been the main alternative for plasma protein biomarker discovery [44]. However, the two approaches are complementary. While MS-based methods are biased to quantify the most abundant plasma proteins, that method gives direct evidence for proteins through the specific mass spectra of the protein or peptide [45]. In contrast, when using OLINK and other antibody-based platforms, defined protein groups are quantified in targeted analyses, including proteins with low concentrations [46].

In conclusion, in this nested case–control study, elevated levels of ADGRE2 and Metrnl prior to diagnosis suggested activation of the innate immune system, possibly through macrophage and neutrophil differentiation and activation. The association between ROR1 levels and subsequent GCA might reflect an early inflammatory process. Furthermore, the protective role of FBP1 needs to be further investigated.

## Supplementary Material

keae073_Supplementary_Data

## Data Availability

The key data underlying this article will be shared on reasonable request to the corresponding author.
