# Supplementary material for: Associations between plasma metabolism-associated proteins and future development of giant cell arteritis: results from a prospective study
Source: Rheumatology (Oxford). 2024 Feb 3;64(2):714–21. doi: 10.1093/rheumatology/keae073 (PMC11781587; doi:10.1093/rheumatology/keae073)

**Supplementary material**

**Supplementary Table S1. Investigated proteins.**

|  |  | **UniProt No** |
| --- | --- | --- |
| **AHCY** | Adenosylhomocysteinase | [P23526](https://www.uniprot.org/uniprotkb/P23526/entry) |
| **ADGRE2** | Adhesion G protein-coupled receptor E2 | [Q9UHX3](https://www.uniprot.org/uniprotkb/Q9UHX3/entry) |
| **ADGRG2** | Adhesion G-protein coupled receptor G2 | [Q8IZP9](https://www.uniprot.org/uniprotkb/Q8IZP9/entry) |
| **APLP1** | Amyloid-like protein 1 | **P51693** |
| **ANGPT2** | Angiopoietin-2 | [O15123](https://www.uniprot.org/uniprotkb/O15123/entry) |
| **ANGPTL1** | Angiopoietin-related protein 1 | [O95841](https://www.uniprot.org/uniprotkb/O95841/entry) |
| **ANGPTL7** | Angiopoietin-related protein 7 | [O43827](https://www.uniprot.org/uniprotkb/O43827/entry) |
| **ANXA11** | Annexin A11 | [P50995](https://www.uniprot.org/uniprotkb/P50995/entry) |
| **ANXA4** | Annexin A4 | [P09525](https://www.uniprot.org/uniprotkb/P09525/entry) |
| **GHRL** | Appetite-regulating hormone | [Q9UBU3](https://www.uniprot.org/uniprotkb/Q9UBU3/entry) |
| **ARG1** | Arginase-1 | [P05089](https://www.uniprot.org/uniprotkb/P05089/entry) |
| **DDC** | Aromatic-L-amino-acid decarboxylase | [P20711](https://www.uniprot.org/uniprotkb/P20711/entry) |
| **CD79B** | B-cell antigen receptor complex-associated protein beta | [P40259](https://www.uniprot.org/uniprotkb/P40259/entry) |
| **CDH2** | Cadherin-2 | [P19022](https://www.uniprot.org/uniprotkb/P19022/entry) |
| **CDHR5** | Cadherin-related family member 5 | [Q9HBB8](https://www.uniprot.org/uniprotkb/Q9HBB8/entry) |
| **CLSTN2** | Calsyntenin-2 | [Q9H4D0](https://www.uniprot.org/uniprotkb/Q9H4D0/entry) |
| **CA13** | Carbonic anhydrase 13 | [Q8N1Q1](https://www.uniprot.org/uniprotkb/Q8N1Q1/entry) |
| **COMT** | Catechol O-methyltransferase | [P21964](https://www.uniprot.org/uniprotkb/P21964/entry) |
| **CTSO** | Cathepsin O | [P43234](https://www.uniprot.org/uniprotkb/P43234/entry) |
| **CD2AP** | CD2-associated protein | [Q9Y5K6](https://www.uniprot.org/uniprotkb/Q9Y5K6/entry) |
| **CHRDL2** | Chordin-like protein 2 | [Q6WN34](https://www.uniprot.org/uniprotkb/Q6WN34/entry) |
| **CLUL1** | Clusterin-like protein 1 | [Q15846](https://www.uniprot.org/uniprotkb/Q15846/entry) |
| **CCDC80** | Coiled-coil domain-containing protein 80 | [Q76M96](https://www.uniprot.org/uniprotkb/Q76M96/entry) |
| **CRKL** | Crk-like protein | [P46109](https://www.uniprot.org/uniprotkb/P46109/entry) |
| **CLEC5A** | C-type lectin domain family 5 member A | [Q9NY25](https://www.uniprot.org/uniprotkb/Q9NY25/entry) |
| **CLMP** | CXADR-like membrane protein | [Q9H6B4](https://www.uniprot.org/uniprotkb/Q9H6B4/entry) |
| **DIABLO** | Diablo homolog, mitochondrial | [Q9NR28](https://www.uniprot.org/uniprotkb/Q9NR28/entry) |
| **QDPR** | Dihydropteridine reductase | [P09417](https://www.uniprot.org/uniprotkb/P09417/entry) |
| **DPP7** | Dipeptidyl peptidase 2 | [Q9UHL4](https://www.uniprot.org/uniprotkb/Q9UHL4/entry) |
| **DAB2** | Disabled homolog 2 | [P98082](https://www.uniprot.org/uniprotkb/P98082/entry) |
| **APEX1** | DNA-(apurinic or apyrimidinic site) lyase | [Q5TZP7](https://www.uniprot.org/uniprotkb/Q5TZP7/entry) |
| **ENTPD5** | Ectonucleoside triphosphate diphosphohydrolase | [O75356](https://www.uniprot.org/uniprotkb/O75356/entry) |
| **ENPP7** | Ectonucleotide pyrophosphatase/phosphodiesterase family member 7 | [Q6UWV6](https://www.uniprot.org/uniprotkb/Q6UWV6/entry) |
| **RNASE3** | Eosinophil cationic protein | [P12724](https://www.uniprot.org/uniprotkb/P12724/entry) |
| **FCRL1** | Fc receptor-like protein 1 | [Q96LA6](https://www.uniprot.org/uniprotkb/Q96LA6/entry) |
| **FBP1** | Fructose-1,6-bisphosphatase 1 | [P09467](https://www.uniprot.org/uniprotkb/P09467/entry) |
| **GAL** | Galanin peptides | [P22466](https://www.uniprot.org/uniprotkb/P22466/entry) |
| **ENO2** | Gamma-enolase | [P09104](https://www.uniprot.org/uniprotkb/P09104/entry) |
| **GLRX** | Glutaredoxin-1 | [P35754](https://www.uniprot.org/uniprotkb/P35754/entry) |
| **GRAP2** | GRB2-related adapter protein 2 | [O75791](https://www.uniprot.org/uniprotkb/O75791/entry) |
| **HDGF** | Hepatoma-derived growth | [P51858](https://www.uniprot.org/uniprotkb/P51858/entry) |
| **ROR1** | inactive tyrosine-protein kinase transmembrane receptor | [Q01973](https://www.uniprot.org/uniprotkb/Q01973/entry) |
| **IGFBPL1** | Insulin-like growth factor-binding protein-like 1 | [Q8WX77](https://www.uniprot.org/uniprotkb/Q8WX77/entry) |
| **ITGB7** | Integrin beta-7 | [P26010](https://www.uniprot.org/uniprotkb/P26010/entry) |
| **KLK10** | Kallikrein-10 | [O43240](https://www.uniprot.org/uniprotkb/O43240/entry) |
| **KYAT1** | Kynurenine--oxoglutarate transaminase 1 | [Q16773](https://www.uniprot.org/uniprotkb/Q16773/entry) |
| **BAG6** | Large proline-rich protein | [P46379](https://www.uniprot.org/uniprotkb/P46379/entry) |
| **LRIG1** | Leucine-rich repeats and immunoglobulin-like domains protein 1 | [Q96JA1](https://www.uniprot.org/uniprotkb/Q96JA1/entry) |
| **LILRA5** | Leukocyte immunoglobulin-like receptor subfamily A member | [A6NI73](https://www.uniprot.org/uniprotkb/A6NI73/entry) |
| **LRP11** | Low-density lipoprotein receptor-related protein 11 | [Q86VZ4](https://www.uniprot.org/uniprotkb/Q86VZ4/entry) |
| **ACP6** | Lysophosphatidic acid phosphatase type 6 | [Q9NPH0](https://www.uniprot.org/uniprotkb/Q9NPH0/entry) |
| **MEP1B** | Meprin A subunit beta | [Q16820](https://www.uniprot.org/uniprotkb/Q16820/entry) |
| **METRNL** | Meteorin-like protein | [Q641Q3](https://www.uniprot.org/uniprotkb/Q641Q3/entry) |
| **MCFD2** | Multiple coagulation factor deficiency protein 2 | [Q8NI22](https://www.uniprot.org/uniprotkb/Q8NI22/entry) |
| **NADK** | NAD kinase | [O95544](https://www.uniprot.org/uniprotkb/O95544/entry) |
| **NECTIN2** | Nectin-2 | [Q92692](https://www.uniprot.org/uniprotkb/Q92692/entry) |
| **NPDC1** | Neural proliferation differentiation and control protein 1 | [Q9NQX5](https://www.uniprot.org/uniprotkb/Q9NQX5/entry) |
| **NPTXR** | Neuronal pentraxin receptor | [O95502](https://www.uniprot.org/uniprotkb/O95502/entry) |
| **NOMO1** | Nodal modulator 1 | [Q15155](https://www.uniprot.org/uniprotkb/Q15155/entry) |
| **NT-proBNP** | N-terminal prohormone of brain natriuretic peptide | [P01160](https://www.uniprot.org/uniprotkb/P01160/entry) |
| **PILRB** | Paired immunoglobulin-like type 2 receptor beta | [Q9UKJ0](https://www.uniprot.org/uniprotkb/Q9UKJ0/entry) |
| **FKBP4** | Peptidyl-prolyl cis-trans isomerase FKBP4 | [Q02790](https://www.uniprot.org/uniprotkb/Q02790/entry) |
| **PAG1** | Phosphoprotein associated with glycosphingolipidenriched microdomains 1 | [Q9NWQ8](https://www.uniprot.org/uniprotkb/Q9NWQ8/entry) |
| **CTSH** | Pro-cathepsin H | [P09668](https://www.uniprot.org/uniprotkb/P09668/entry) |
| **FAM3C** | Protein FAM3C | [Q92520](https://www.uniprot.org/uniprotkb/Q92520/entry) |
| **PPP1R2** | Protein phosphatase inhibitor 2 | [P41236](https://www.uniprot.org/uniprotkb/P41236/entry) |
| **S100P** | Protein S100-P | [P25815](https://www.uniprot.org/uniprotkb/P25815/entry) |
| **REG4** | Regenerating islet-derived protein 4 | [Q9BYZ8](https://www.uniprot.org/uniprotkb/Q9BYZ8/entry) |
| **RTN4R** | Reticulon-4 receptor | [Q9BZR6](https://www.uniprot.org/uniprotkb/Q9BZR6/entry) |
| **ALDH1A1** | Retinal dehydrogenase 1 | [P00352](https://www.uniprot.org/uniprotkb/P00352/entry) |
| **NQO2** | Ribosyldihydronicotinamide dehydrogenase [quinone] | [P16083](https://www.uniprot.org/uniprotkb/P16083/entry) |
| **SSC4D** | Scavenger receptor cysteine-rich domain-containing group B protein | [Q8WTU2](https://www.uniprot.org/uniprotkb/Q8WTU2/entry) |
| **SOST** | Sclerostin | [Q9BQB4](https://www.uniprot.org/uniprotkb/Q9BQB4/entry) |
| **SEMA3F** | Semaphorin-3F | [Q13275](https://www.uniprot.org/uniprotkb/Q13275/entry) |
| **SERPINB6** | Serpin B6 | [P35237](https://www.uniprot.org/uniprotkb/P35237/entry) |
| **SERPINB8** | Serpin B8 | [P50452](https://www.uniprot.org/uniprotkb/P50452/entry) |
| **SIGLEC7** | Sialic acid-binding Ig-like lectin 7 | [Q9Y286](https://www.uniprot.org/uniprotkb/Q9Y286/entry) |
| **CD164** | Sialomucin core protein 24 | [Q04900](https://www.uniprot.org/uniprotkb/Q04900/entry) |
| **CANT1** | Soluble calcium-activated nucleotidase 1 | [Q8WVQ1](https://www.uniprot.org/uniprotkb/Q8WVQ1/entry) |
| **SUMF2** | Sulfatase-modifying factor 2 | [Q8NBJ7](https://www.uniprot.org/uniprotkb/Q8NBJ7/entry) |
| **SNAP23** | Synaptosomal-associated protein 23 | [O00161](https://www.uniprot.org/uniprotkb/O00161/entry) |
| **SDC4** | Syndecan-4 | [P31431](https://www.uniprot.org/uniprotkb/P31431/entry) |
| **CD1C** | T-cell surface glycoprotein CD1c | [P29017](https://www.uniprot.org/uniprotkb/P29017/entry) |
| **THOP1** | Thimet oligopeptidase | [P52888](https://www.uniprot.org/uniprotkb/P52888/entry) |
| **TXNDC5** | Thioredoxin domain-containing protein 5 | [Q8NBS9](https://www.uniprot.org/uniprotkb/Q8NBS9/entry) |
| **TYMP** | Thymidine phosphorylase | [P19971](https://www.uniprot.org/uniprotkb/P19971/entry) |
| **TSHB** | Thyrotropin subunit beta | [P01222](https://www.uniprot.org/uniprotkb/P01222/entry) |
| **TFF2** | Trefoil factor 2 | [Q03403](https://www.uniprot.org/uniprotkb/Q03403/entry) |
| **TINAGL1** | Tubulointerstitial nephritis antigen-like | [Q9GZM7](https://www.uniprot.org/uniprotkb/Q9GZM7/entry) |
| **TYRO3** | Tyrosine-protein kinase receptor TYRO3 | [Q06418](https://www.uniprot.org/uniprotkb/Q06418/entry) |
| **USP8** | Ubiquitin carboxyl-terminal hydrolase 8 | [P40818](https://www.uniprot.org/uniprotkb/P40818/entry) |
| **VCAN** | Versican core protein | [P13611](https://www.uniprot.org/uniprotkb/P13611/entry) |

**Supplementary Table S2. A priori hypotheses**

| **Biomarker** | **Hypothesis** |
| --- | --- |
| METRNL | Elevated in pre-GCA cases |
| FBP1 | Elevated in pre-GCA cases |
| GAL | Elevated in pre-GCA cases |
| GHRL | Reduced in pre-GCA cases |
| ADGRE2 | Elevated in pre-GCA cases |
| NECTIN2 | Elevated in pre-GCA cases |

**Supplementary Table S3. Components identified in principal component analysis, with factor loading of individual proteins**

|  | Factor 1 | Factor 2 | Factor 3 | Factor 4 | Factor 5 | Factor 6 | Factor 7 |
| --- | --- | --- | --- | --- | --- | --- | --- |
| Eigenvalue | 26.28 | 9.53 | 3.90 | 3.88 | 2.56 | 2.29 | 2.03 |
| METRNL | 0.11 | 0.78 | -0.03 | -0.10 | 0.14 | 0.03 | -0.04 |
| FBP1 | 0.04 | 0.18 | -0.003 | **0.78** | 0.04 | 0.07 | 0.03 |
| GAL | -0.07 | 0.15 | 0.04 | -0.07 | -0.02 | 0.03 | 0.06 |
| GHRL | 0.03 | 0.20 | 0.06 | -0.15 | 0.16 | -0.05 | 0.12 |
| ADGRE2 | 0.12 | 0.61 | 0.19 | -0.03 | 0.11 | 0.09 | 0.10 |
| NECTIN2 | 0.42 | **0.72** | 0.10 | 0.03 | -0.03 | 0.12 | 0.00 |
| AHCY | 0.47 | 0.03 | 0.21 | 0.45 | 0.06 | 0.25 | -0.22 |
| FCRL1 | 0.01 | 0.46 | 0.40 | -0.02 | 0.24 | 0.04 | 0.21 |
| ANGPTL7 | 0.12 | 0.51 | 0.12 | 0.13 | 0.14 | 0.22 | 0.18 |
| APEX1 | 0.37 | 0.10 | 0.65 | 0.11 | -0.02 | 0.03 | -0.02 |
| TXNDC5 | 0.66 | 0.42 | 0.21 | 0.03 | 0.06 | 0.09 | -0.02 |
| PPP1R2 | 0.65 | 0.37 | 0.05 | -0.04 | -0.05 | 0.03 | 0.003 |
| CLMP | 0.04 | **0.78** | -0.01 | 0.05 | 0.001 | -0.12 | -0.10 |
| LRIG1 | 0.02 | 0.43 | -0.06 | 0.08 | 0.24 | -0.05 | 0.04 |
| NPTXR | 0.01 | 0.54 | 0.05 | -0.02 | 0.30 | 0.16 | 0.16 |
| THOP1 | 0.39 | 0.26 | 0.46 | 0.15 | -0.20 | 0.04 | 0.32 |
| CTSO | 0.12 | 0.68 | 0.12 | 0.16 | -0.12 | -0.01 | 0.08 |
| CD164 | 0.64 | 0.49 | 0.17 | 0.07 | 0.04 | 0.008 | 0.22 |
| DDC | 0.09 | 0.14 | -0.04 | 0.01 | 0.16 | 0.03 | 0.02 |
| ACP6 | 0.32 | 0.33 | 0.16 | -0.04 | 0.01 | 0.004 | 0.07 |
| TFF2 | 0.04 | 0.29 | 0.01 | 0.04 | -0.03 | 0.10 | 0.08 |
| S100P | 0.24 | 0.11 | 0.23 | 0.16 | 0.06 | 0.62 | 0.04 |
| ANGPT2 | 0.11 | 0.40 | 0.08 | -0.20 | 0.12 | 0.29 | -0.05 |
| CD2AP | 0.41 | 0.03 | **0.74** | 0.12 | -0.02 | -0.05 | 0.17 |
| CLEC5A | 0.10 | 0.51 | 0.06 | 0.03 | 0.21 | 0.14 | 0.20 |
| TINAGL1 | 0.09 | 0.57 | 0.05 | -0.10 | 0.20 | 0.17 | -0.02 |
| GLRX | 0.27 | 0.11 | 0.41 | 0.63 | 0.01 | 0.19 | 0.07 |
| ENO2 | **0.84** | 0.11 | 0.16 | 0.34 | 0.07 | 0.01 | 0.06 |
| NADK | 0.17 | 0.23 | 0.36 | 0.03 | 0.08 | 0.10 | -0.10 |
| SERPINB8 | 0.20 | 0.31 | 0.18 | -0.07 | -0.02 | -0.003 | 0.42 |
| SERPINB6 | **0.89** | 0.32 | 0.03 | 0.06 | 0.04 | 0.07 | 0.08 |
| CDHR5 | 0.03 | 0.34 | 0.03 | 0.08 | -0.24 | -0.05 | 0.14 |
| CCDC80 | 0.08 | **0.72** | -0.07 | -0.10 | 0.03 | -0.14 | 0.04 |
| DIABLO | **0.83** | 0.09 | 0.08 | -0.19 | 0.04 | 0.13 | 0.10 |
| CA13 | **0.90** | 0.09 | -0.10 | 0.21 | 0.05 | 0.005 | 0.02 |
| SEMA3F | 0.25 | **0.76** | 0.04 | 0.09 | -0.07 | 0.24 | 0.12 |
| KLK10 | 0.09 | 0.55 | -0.09 | 0.03 | 0.19 | 0.11 | 0.15 |
| PILRB | 0.21 | 0.52 | -0.05 | 0.13 | -0.08 | 0.09 | 0.02 |
| ANGPTL1 | 0.13 | 0.35 | 0.03 | 0.23 | 0.49 | 0.02 | 0.07 |
| APLP1 | -0.07 | 0.34 | -0.05 | -0.15 | 0.28 | 0.07 | 0.27 |
| ADGRG2 | 0.10 | 0.32 | 0.17 | 0.12 | 0.44 | 0.14 | 0.46 |
| TYMP | 0.57 | 0.22 | 0.04 | 0.40 | 0.06 | -0.07 | -0.11 |
| GRAP2 | **0.91** | 0.09 | 0.12 | 0.02 | -0.04 | 0.08 | 0.03 |
| LILRA5 | 0.14 | 0.51 | 0.03 | 0.10 | -0.16 | 0.12 | 0.16 |
| ALDH1A1 | 0.22 | 0.22 | 0.40 | 0.61 | -0.19 | 0.08 | -0.005 |
| CD79B | 0.11 | 0.69 | 0.26 | -0.12 | 0.01 | 0.06 | 0.06 |
| ANXA11 | **0.87** | 0.03 | 0.16 | -0.20 | -0.03 | 0.09 | 0.04 |
| SIGLEC7 | 0.17 | 0.51 | 0.17 | 0.12 | -0.03 | 0.13 | 0.30 |
| ITGB7 | 0.19 | 0.18 | 0.04 | 0.16 | 0.07 | 0.70 | 0.13 |
| QDPR | **0.78** | 0.25 | 0.25 | 0.31 | -0.12 | -0.09 | 0.05 |
| SNAP23 | **0.94** | 0.06 | 0.08 | 0.12 | -0.03 | 0.06 | 0.03 |
| ENTPD5 | 0.001 | 0.26 | 0.14 | 0.02 | 0.45 | 0.12 | 0.10 |
| CLSTN2 | 0.08 | 0.40 | 0.09 | -0.06 | 0.19 | 0.11 | 0.12 |
| COMT | **0.89** | 0.05 | 0.05 | -0.11 | -0.07 | -0.03 | -0.05 |
| CLUL1 | -0.04 | 0.18 | -0.05 | 0.08 | 0.67 | 0.10 | 0.15 |
| HDGF | 0.32 | 0.10 | **0.80** | 0.04 | -0.04 | 0.08 | -0.11 |
| CHRDL2 | 0.04 | 0.22 | -0.01 | -0.11 | -0.04 | -0.06 | 0.05 |
| CTSH | 0.02 | 0.13 | -0.01 | 0.12 | -0.09 | 0.00 | 0.08 |
| NOMO1 | 0.42 | 0.45 | 0.08 | 0.22 | -0.14 | 0.03 | 0.13 |
| NQO2 | 0.07 | 0.14 | -0.03 | 0.006 | -0.12 | 0.40 | -0.07 |
| SOST | 0.19 | 0.43 | 0.15 | -0.05 | -0.12 | 0.29 | -0.02 |
| FAM3C | 0.22 | **0.82** | 0.17 | 0.17 | 0.007 | 0.03 | 0.10 |
| DPP7 | 0.58 | 0.14 | -0.02 | -0.02 | 0.11 | -0.05 | -0.07 |
| LRP11 | 0.17 | **0.81** | 0.10 | 0.10 | -0.04 | 0.005 | 0.14 |
| ENPP7 | -0.02 | 0.18 | 0.02 | 0.07 | -0.002 | 0.04 | -0.01 |
| SSC4D | 0.06 | 0.14 | 0.13 | 0.16 | -0.63 | 0.05 | 0.02 |
| MCFD2 | 0.70 | 0.27 | 0.15 | -0.04 | 0.08 | 0.02 | -0.05 |
| REG4 | 0.15 | 0.52 | 0.001 | -0.04 | 0.07 | 0.11 | 0.09 |
| SUMF2 | 0.50 | 0.42 | 0.23 | 0.02 | 0.06 | -0.02 | -0.05 |
| CANT1 | 0.28 | **0.72** | 0.20 | 0.15 | 0.007 | 0.09 | 0.19 |
| CD1C | 0.06 | 0.25 | -0.07 | -0.02 | 0.14 | 0.05 | 0.76 |
| CDH2 | 0.20 | 0.62 | -0.02 | 0.15 | -0.17 | -0.05 | -0.02 |
| TYRO3 | 0.12 | 0.48 | -0.02 | 0.16 | 0.12 | 0.18 | 0.45 |
| CRKL | **0.95** | 0.10 | 0.08 | 0.03 | -0.04 | -0.04 | 0.02 |
| IGFBPL1 | 0.18 | **0.72** | 0.07 | 0.07 | 0.04 | 0.11 | -0.08 |
| RTN4R | 0.07 | 0.41 | 0.08 | 0.11 | -0.47 | 0.08 | 0.04 |
| VCAN | 0.09 | 0.42 | 0.32 | 0.19 | 0.28 | 0.28 | 0.17 |
| TSHB | 0.09 | 0.11 | -0.05 | 0.00 | -0.04 | 0.004 | -0.04 |
| BAG6 | 0.52 | 0.02 | 0.34 | -0.04 | 0.40 | -0.03 | 0.14 |
| USP8 | 0.34 | 0.12 | 0.57 | 0.02 | 0.01 | 0.52 | -0.001 |
| FKBP4 | 0.34 | 0.17 | 0.69 | 0.13 | 0.005 | 0.12 | -0.05 |
| SDC4 | 0.51 | -0.03 | 0.11 | 0.25 | 0.14 | -0.02 | 0.002 |
| PAG1 | 0.68 | 0.33 | 0.20 | -0.04 | 0.05 | 0.18 | 0.23 |
| KYAT1 | 0.68 | 0.12 | 0.33 | 0.14 | 0.04 | -0.10 | -0.01 |
| DAB2 | **0.79** | 0.19 | 0.09 | 0.18 | -0.08 | 0.22 | -0.02 |
| NPDC1 | 0.22 | **0.85** | 0.10 | 0.12 | -0.01 | -0.04 | -0.005 |
| MEP1B | 0.06 | 0.11 | -0.004 | 0.09 | 0.02 | 0.08 | -0.02 |
| ROR1 | 0.09 | **0.72** | 0.15 | 0.06 | 0.28 | 0.09 | 0.24 |
| NTproBNP | 0.09 | 0.41 | 0.03 | -0.08 | 0.39 | -0.20 | -0.18 |
| RNASE3 | 0.10 | 0.12 | 0.14 | -0.08 | -0.08 | 0.07 | 0.12 |
| ANXA4 | **0.80** | -0.03 | 0.16 | -0.11 | -0.02 | 0.35 | 0.02 |
| ARG1 | 0.009 | 0.006 | 0.44 | 0.44 | 0.09 | 0.24 | 0.18 |

**Supplementary Table S4. Proportions with self-reported history of comorbidities, at time of screening**

|  | Cases (N=94) | Controls (N=97) |
| --- | --- | --- |
| Malignancies | 6/71 (8.3 %) | 11/93 (11.8 %) |
| Diabetes | 2/72 (2.8 %) | 4/94 (4.3 %) |
| Myocardial infarction | 3/72 (4.2 %) | 2/93 (2.2 %) |
| Stroke | 1/71 (1.4 %) | 2/93 (2.2 %) |

**Supplementary Table S5. Proportions with self-reported medication, at time of screening**

|  | Cases (N=94) | Controls (N=97) |
| --- | --- | --- |
| Lipid lowering drugs | 1 (1 %) | 8 (8 %) |
| Antidiabetics | 0 (0 %) | 3 (3 %) |
| Calcium blockers | 5 (5 %) | 4 (4 %) |
| ACE inhibitors | 0 (0 %) | 1 (1 %) |
| Beta blockers | 12 (13 %) | 13 (13 %) |
| Diuretics | 7 (7 %) | 6 (6 %) |
| Glucocorticoids* | 0 (0 %) | 0 (0 %) |

*Data missing for 17 controls and 28 cases

**Supplementary Figure S1. Scree plot of components identified in the principal component analysis, highest to lowest Eigenvalue.**


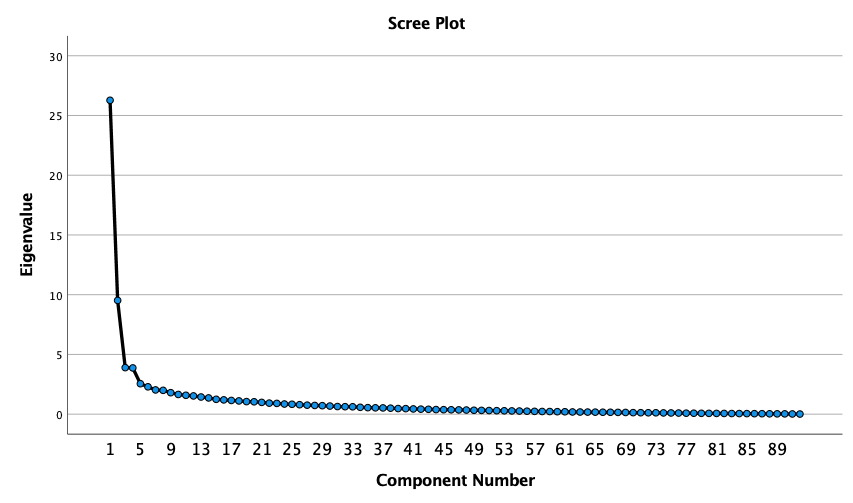

Supplement: keae073_Supplementary_Data [file keae073_supplementary_data.doc]
